# Activation of cannabinoid type 1 receptor (CB1) modulates oligodendroglial process branching complexity in rat hippocampal cultures stimulated by olfactory ensheathing glia-conditioned medium

**DOI:** 10.3389/fncel.2023.1134130

**Published:** 2023-04-17

**Authors:** Yolanda Paes-Colli, Priscila M. P. Trindade, Louise C. Vitorino, Fabiana Piscitelli, Fabio Arturo Iannotti, Raquel M. P. Campos, Alinny R. Isaac, Andrey Fabiano Lourenço de Aguiar, Silvana Allodi, Fernando G. de Mello, Marcelo Einicker-Lamas, Raphael de Siqueira-Santos, Vincenzo Di Marzo, Bakhos A. Tannous, Litia A. Carvalho, Ricardo A. De Melo Reis, Luzia S. Sampaio

**Affiliations:** ^1^Laboratório de Neuroquímica, Instituto de Biofísica Carlos Chagas Filho, Universidade Federal do Rio de Janeiro, Rio de Janeiro, Brazil; ^2^Laboratório de Neurobiologia Comparativa e do Desenvolvimento, Instituto de Biofísica Carlos Chagas Filho, Universidade Federal do Rio de Janeiro, Rio de Janeiro, Brazil; ^3^Endocannabinoid Research Group, Istituto di Chimica Biomolecolare, CNR, Pozzuoli, Italy; ^4^Laboratório de Doenças Neurodegenerativas, Instituto de Bioquímica Médica Leopoldo de Meis, Universidade Federal do Rio de Janeiro, Rio de Janeiro, Brazil; ^5^Laboratório de Biomembranas, Instituto de Biofísica Carlos Chagas Filho, Universidade Federal do Rio de Janeiro, Rio de Janeiro, Brazil; ^6^Laboratório de Agregação de Proteínas e Amiloidoses, Instituto de Bioquímica Médica Leopoldo de Meis, Universidade Federal do Rio de Janeiro, Rio de Janeiro, Brazil; ^7^Canada Excellence Research Chair on the Microbiome-Endocannabinoidome Axis, Laval University, Quebec, QC, Canada; ^8^Experimental Therapeutics and Molecular Imaging Laboratory, Massachusetts General Hospital, Boston, MA, United States; ^9^Neuroscience Program, Harvard Medical School, Boston, MA, United States

**Keywords:** cannabinoid receptor 1, oligodendrocyte, olfactory ensheathing glia, endocannabinoids, myelin basic protein (MBP), hippocampus

## Abstract

The endocannabinoid system (ECS) refers to a complex cell-signaling system highly conserved among species formed by numerous receptors, lipid mediators (endocannabinoids) and synthetic and degradative enzymes. It is widely distributed throughout the body including the CNS, where it participates in synaptic signaling, plasticity and neurodevelopment. Besides, the olfactory ensheathing glia (OEG) present in the olfactory system is also known to play an important role in the promotion of axonal growth and/or myelination. Therefore, both OEG and the ECS promote neurogenesis and oligodendrogenesis in the CNS. Here, we investigated if the ECS is expressed in cultured OEG, by assessing the main markers of the ECS through immunofluorescence, western blotting and qRT-PCR and quantifying the content of endocannabinoids in the conditioned medium of these cells. After that, we investigated whether the production and release of endocannabinoids regulate the differentiation of oligodendrocytes co-cultured with hippocampal neurons, through Sholl analysis in oligodendrocytes expressing O4 and MBP markers. Additionally, we evaluated through western blotting the modulation of downstream pathways such as PI3K/Akt/mTOR and ERK/MAPK, being known to be involved in the proliferation and differentiation of oligodendrocytes and activated by CB1, which is the major endocannabinoid responsive receptor in the brain. Our data show that OEG expresses key genes of the ECS, including the CB1 receptor, FAAH and MAGL. Besides, we were able to identify AEA, 2-AG and AEA related mediators palmitoylethanolamide (PEA) and oleoylethanolamide (OEA), in the conditioned medium of OEG cultures. These cultures were also treated with URB597 10-9 M, a FAAH selective inhibitor, or JZL184 10-9 M, a MAGL selective inhibitor, which led to the increase in the concentrations of OEA and 2-AG in the conditioned medium. Moreover, we found that the addition of OEG conditioned medium (OEGCM) enhanced the complexity of oligodendrocyte process branching in hippocampal mixed cell cultures and that this effect was inhibited by AM251 10-6 M, a CB1 receptor antagonist. However, treatment with the conditioned medium enriched with OEA or 2-AG did not alter the process branching complexity of premyelinating oligodendrocytes, while decreased the branching complexity in mature oligodendrocytes. We also observed no change in the phosphorylation of Akt and ERK 44/42 in any of the conditions used. In conclusion, our data show that the ECS modulates the number and maturation of oligodendrocytes in hippocampal mixed cell cultures.

## 1. Introduction

Demyelinating neurodegenerative diseases such as multiple sclerosis and leukodystrophies are characterized by the progressive loss of myelin and impaired axonal conduction, leading to mortality and morbidity (Jaramillo-Merchan et al., 2013). The loss of oligodendrocytes (OLs), which are the cells responsible for producing myelin in the central nervous system (CNS), is a hallmark of these diseases (Lopez Juarez et al., 2016). OLs are derived from oligodendrocyte precursor cells (OPCs), multipotent cells derived from neural stem cells (NSCs) during neurogenesis. This process occurs throughout the lifespan, at a higher rate during CNS development, and at a lower rate during adulthood, mostly in the subventricular zone (SVZ) and subgranular zone (SGZ) of the hippocampus (Tognatta and Miller, 2016). Developing novel therapeutic strategies to generate and maintain OLs and possibly halt their death is crucial to circumvent the demyelination caused by neurodegenerative conditions and/or CNS trauma.

The Endocannabinoid System (ECS) is a highly conserved neuromodulatory system that plays an important role in the central nervous system (CNS) (Elphick, 2012; Zou and Kumar, 2018). Although the number of endocannabinoids and endocannabinoid-like molecules is still growing, the “on-demand” synthesis of the major endocannabinoids anandamide (AEA) and 2-arachidonoylglycerol (2-AG) followed by the activation of the cannabinoid receptors of type 1 (CB1) and type 2 (CB2) remain the foremost mechanism through which the ECS exert its functions (Iannotti et al., 2016; Di Marzo, 2018). The ECS plays critical functions in the development of the activity of the nervous system (Di Marzo et al., 1998; Ruiz-Contreras et al., 2022). Remarkably, among such functions, the ECS is known to regulate OL proliferation and differentiation (Gomez et al., 2011). To this regard, Gomez et al. demonstrated that the pharmacological blockade of CB1 by WIN55,212-2 enhances *in vivo* oligodendrogenesis after a CNS injury and that this effect is similar to that of AEA (Solbrig et al., 2010) or 2-AG (Gomez et al., 2010). The same investigators have further demonstrated that the activation of PI3K/Akt and mTOR signaling pathways following the activation of CB1 and CB2 is necessary to promote OL differentiation *in vitro* (Gomez et al., 2011).

The olfactory ensheathing glia (OEG) is an essential differentiated stem cell-like glial cell that enwraps the axons of sensory olfactory receptors as they grow from the olfactory epithelium into the olfactory bulb. OEGs migrate from the peripheral nervous system (PNS) to the central nervous system (CNS), a critical process for the development and maintenance of the olfactory system and axonal extension during the physiological neuronal turnover during adulthood (Tsai et al., 2017). OEG secrete molecules known to induce the survival of neural cells and clearance of debris in several models of CNS lesions and neurodegenerative diseases (Franssen et al., 2007; Pastrana et al., 2007; Su et al., 2013; Gu et al., 2017; Li et al., 2017). Several studies have shown the therapeutic potential of OEG and their conditioned medium in studies involving regeneration and cancer (Ramón-Cueto and Nieto-Sampedro, 1994; Gómez-Pinedo et al., 2011; Azimi Alamouti et al., 2015; Goulart et al., 2016; Gu et al., 2017; Carvalho et al., 2018). Therefore, by taking advantage of OEG unique properties, we explored the potential use and efficacy of the olfactory ensheathing glia-conditioned medium (OEGCM) in inducing OL proliferation in various stages of maturation (Carvalho et al., 2014). Notably, the effect of OEGCM in promoting the proliferation of OL was partially reversed by inhibitors of ERK/MAPK, p38MAPK and PI3K/Akt/mTOR signaling pathways.

Moreover, we explored whether OEGs could change the endocannabinoid system activity and how these changes could impact OEGCM-induced proliferation and morphologic differentiation of OLs co-cultured with hippocampal progenitor cells.

## 2. Materials and methods

### 2.1. Animals

The experiments involving animals were approved by and carried out in accordance with the guidelines of the Ethics Committee for the Use of Animals in Research of the Federal University of Rio Janeiro (CEUA IBCCF protocols #020, #102/19 and #067/21), and in accordance with the guidelines of the Brazilian Society of Neuroscience and Behavior (SBNeC). The animals were maintained in a 12h/12h light cycle and controlled temperature (23 ± 1 °C) with access to water and food *ad libitum*.

### 2.2. Primary cell cultures from OEG and OEGCM

OEGs primary cell cultures were harvested from the olfactory bulb (OB) of adult Wistar rats (3-4 months) as previously reported (Carvalho et al., 2014), and at least 20 animals were used in this study. The OEG primary cell culture was also performed following a protocol previously described (Nash et al., 2001; Carvalho et al., 2014). Briefly, the olfactory fiber layer from the OB was dissociated and purified by differential adhesion (Nash et al., 2001), and then cells were placed on coverslips, T25 plates and/or 6-well plates previously treated with laminin (40 μg/ml, Sigma Aldrich) in complete DMEM-F12 supplemented with 10% fetal bovine serum and incubated in 5% CO_2_ at 37°C. After the OEG cell culture reached 80% confluence, the OEGCM was collected, filtered (0.22 μm membrane pore), aliquoted and stored at −70°C until use.

For the treatment with the anandamide degradation enzyme inhibitor URB597 (iFAAH) (Cayman Chemical) or 2-AG degradation enzyme inhibitor JZL184 (iMAGL) (Cayman Chemical), OEG cells received every day for 3 days the inhibitors diluted to 10^–9^ M. In the fourth day, the OEGCM was collected, filtered (0.22 μm membrane pore), aliquoted and stored at −70°C until use.

### 2.3. qRT-PCR

RNA was isolated using mini columns (Qiagen) following the instructions of the manufacturer. 500 μg of total RNA was transcribed into cDNA using OneScript^®^ cDNA synthesis kit (Applied Biological Materials). Primer pairs for measuring the expression of CB1, MAGL and FAAH are described in Table 1, and were designed using Primer-BLAST tool (available on NCBI website) using NCBI Reference Sequence and checked against the genebank to avoid cross-reactivity with other known sequences.

**TABLE 1:** Primers pairs sequences used in qRT-PCR.

	Forward primer	Reverse primer
CB1	5′GAAGCCACTCACTCCTGATA AA3′	5′GGCATATACTAGCTCTCCACT TC3′
FAAH	5′CACTTCTCCGGCSTGGTTCT3′	5′CGTGAAACGGCGTTGAGC3 ′
MAGL	5′GTCAACCTCCGACTTGTTCC3′	5′ACCTCTGATCCTTGCCAATC3′
GAPDH	5′GTAACCAGGCGTCCGATAC3′	5′TCTCTGCTCCTCCCTGTTC3′

### 2.4. Quantification of endocannabinoids anandamide (AEA) and 2-arachidonoylglycerol (2-AG) and related mediators, palmitoylethanolamide (PEA) and oleoyl ethanolamide (OEA)

AEA, 2-AG, PEA and OEA levels were analyzed according to the methodology described previously (Iannotti et al., 2013). The OEGCM was collected as already described. From 1 mL of medium, it was obtained the total lipid extract, initially with the addition of 1 mL of methanol and the addition of 2 mL of chloroform on the thawed sample (OEGCM: methanol: chloroform (1:1:2) and sonication at 4°C (1 min) containing internal deuterated standards ([2H]^8^AEA, 5 pmol (Cayman Chemical); and [2H]^5^ 2-AG, [2H]^4^ PEA and [2H]^2^ OEA (Cayman Chemical), 50 pmol each), followed by centrifugation at 2,000 g for 3 min and then, the organic phase was collected, evaporated and freeze-dried in vacuum speed. The final dry weight of the sample was determined and kept stored at −20°C for 48 h.

For the pre-purification, the dried sample was resuspended in chloroform: methanol solution (99:1) and eluted through silica open column chromatography (silica gel 60 HF 254 + 366, Merck). Fractions were obtained by eluting the column with 99:1, 90:10 and 50:50 (v/v) chloroform/methanol. The 90:10 fraction was used for AEA, 2-AG, PEA and OEA quantification by liquid chromatography–atmospheric pressure chemical ionization–mass spectrometry by using a Shimadzu high-performance liquid chromatography apparatus (LC-10ADVP) coupled to a Shimadzu (LCMS-2020) quadrupole mass spectrometry via a Shimadzu atmospheric pressure chemical ionization interface as previously described (Iannotti et al., 2013).

### 2.5. Primary mixed cell cultures from hippocampus

For the hippocampal mixed cell cultures, postnatal Wistar rats (0-2 days) were used, in a total of 80 animals, and each experimental “n” expresses the number of used animals from the same offspring.

The primary mixed cell culture was performed following a protocol previously described (Carvalho et al., 2014). Briefly, the hippocampus was dissected, and the cell suspension was dissociated, filtered, and placed on coverslips and/or 6-well cell plates previously treated with poly-L-lysine (10 μg/ml). Cell cultures were maintained in Neurobasal A medium (Life Technologies), supplemented with 2% B27 (Life Technologies), 2 mM L-glutamine and 1% gentamicin (maintenance medium – NB27). Cells were incubated in 5% CO_2_ at 37°C for 3 days. On day one, cells received OEGCM, where the conditioned medium was diluted in a maintenance medium in a ratio of 1:5 (OEGCM:maintenance medium). For the AM251 (Cayman Chemical) treatment groups, cultures received 10^–6^ M/day. For the cells that received treatments with the OEGCM modulated with enzymes inhibitors, the experimental groups were divided into OEGCM, OEGCM from cells treated with URB597 or OEGCM from cells treated with JZL184, all of them diluted in maintenance medium in the same proportion described before (1:5), incubated at day one and processed in the fourth day after cell plating.

### 2.6. Immunocytochemistry

OEG and hippocampal cell cultures were grown on 13 mm coverslips and fixed for 15 minutes in paraformaldehyde (4%) in phosphate buffer saline (PBS). Coverslips were washed three times with PBS and blocked for 30 min with 1% horse serum + 2% bovine serum albumin (BSA) in PBS with Triton 0.25% (suppressed in O4 staining). The blocking solution was then removed and the primary antibodies of interest were added [P75 (1:100) (#D4B3, Cell Signaling), CB1 (1:100) (SAB2500190, Sigma-Aldrich), MAGL (1:100) (ab152002, Abcam), CNPase (1:100) (SAB4200693, Sigma-Aldrich), Olig2 (1:200) (ab81093, Abcam), O4 (1:300) (produced in hybridoma), MBP (1:100) (sc-13914, Santa Cruz Biotechnology), β-III-tubulin (1:500) (GTX50789, GeneTex)] for overnight incubation at 4°C. Coverslips were washed with PBS and fluorochrome-conjugated secondary antibodies donkey anti-goat Alexa 488, goat anti-mouse Alexa 488 and/or donkey anti-rabbit Alexa 594 (1:400) (Alexa Fluor, Life Technologies) were added for 2h at room temperature protected from light. After rinsing with PBS, coverslips were mounted using a PBS 40% glycerol and DAPI 0.04 μg/ml solution and then examined in a fluorescence microscope (ApoTome 2^®^ - ZEISS).

### 2.7. Western blotting

Cell cultures were washed with PBS, homogenized in lysis buffer (EDTA 1mM, Hepes-Tris 20mM, sucrose 0.25 M, trypsin inhibitor 0.15 mg/ml), the total protein concentration was quantified (Lowry et al., 1951) and separated by polyacrylamide gel electrophoresis (10% SDS-PAGE), and then transferred to nitrocellulose membranes. After 1h in blocking solution (5% low fat dried milk in Tris buffer saline – TBS), immunodetection was performed by incubating the membrane with a specific primary antibody [P75 (1:1,000) (#D4B3, Cell Signaling), CNPase (1:500) (SAB4200693, Sigma-Aldrich), smooth muscle α-actin (1:1,000) (#19245, Cell Signaling), CB1 (1:1,000) (#209550, Calbiochem), pERK 44/42 (1:1,000) (#4376, Cell Signaling), ERK 44/42 (1:1,000) (#9102, Cell Signaling), pAkt 1-2-3 (1:1,000) (sc-7985-R, Santa Cruz Biotechnology) and Akt 1-2-3 (1:1000) (sc-8312, Santa Cruz Biotechnology)] overnight at 4°C. Membranes were washed with TBS-containing 0.05% Tween-20 and incubated with secondary horseradish peroxidase (HRP)-conjugated antibody for 2 h at room temperature. Proteins of interest were finally detected using Immobilon™ Forte (Merck Millipore) and ChemiDoc™ MP Imaging System (Bio-Rad). The same membrane was used for more than one immunodetection, and thus, we performed stripping with NaOH 1M for 1 minute followed by glycine 0.2 M, pH 2.2 for 30 min.

### 2.8. Sholl analysis

Immunocytochemistry anti-O4 and MBP staining of OLs served to assess morphology through size of cell and process branching complexity, using respectively cell diameter and the number of interceptions due to the radium. The diameter of the cell was evaluated as the circumference of the cell measured until the distance of the longest projection. The number of intersections was quantified inside every concentric circle drawn at every 0.5 μm starting from the soma until the periphery of the OL, and the complexity of the OL process branching was evaluated by the area under the curve relating number of intersections in function of distance from the soma. The quantification was done by Sholl analysis (Sholl, 1953) using the ImageJ Sholl Analysis Plugin (NIH, Bethesda, MD, developed by Wayne Rasband) according to the developers’ instructions (Gensel et al., 2010; Falcon-Urrutia et al., 2015).

### 2.9. Statistical analysis

All data were analyzed in terms of normal distribution for proper statistical analysis. For quantification of endocannabinoids and area under the curve of Sholl analysis for O4- and MBP-positive cells after treatment with inhibitors of endocannabinoid degradation enzymes, One-way ANOVA was used, followed by Tukey’s multiple comparison test. For analysis of the diameter and area under the curve of O4- and MBP-positive cells treated with a CB1 antagonist, One-way ANOVA was also used, followed by Šidák’s test. For quantification of total O4-, MBP-, and Olig2-positive cells, distribution was non-parametric, and data were analyzed by Kruskal-Wallis followed by Dunn’s multiple comparison test. Finally, for quantification of Akt and ERK phosphorylation, Friedman was used and followed by Dunn’s test. Statistical significance was considered when p < 0,05.

## 3. Results

### 3.1. Highly purified OEGs culture expresses markers of the endocannabinoid system

**FIGURE 1:**
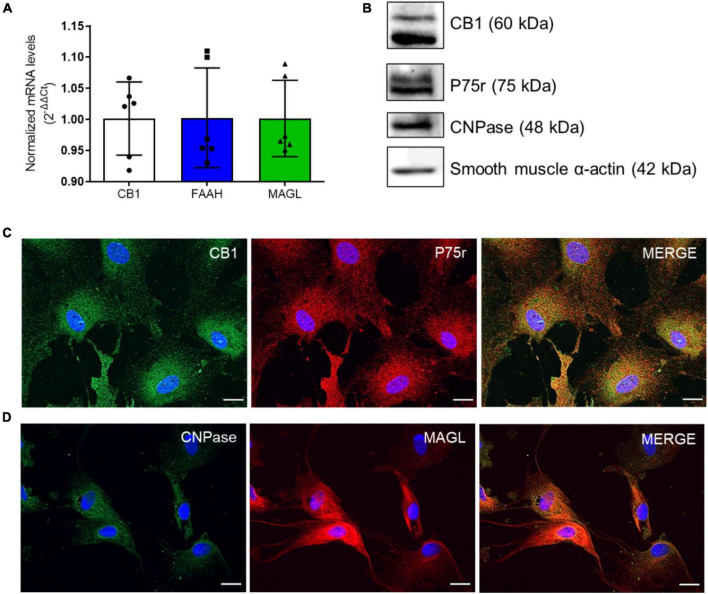
OEG cell cultures express markers of the endocannabinoid system (ECS). In panel **(A)**, CB1, FAAH and MAGL mRNA levels in OEG cells in culture. *N* = 6. In panel **(B)**, western blotting analysis shows the expression of CB1 and the main markers of OEG cell culture (P75r, CNPase and smooth muscle α-actin). Representative image from different experiments. *N* = 3. In panel **(C)**, immunocytochemistry in OEG cell culture for CB1 (in green) and P75r (in red) and in panel **(D)**, CNPase (in green) and MAGL (in red). Cell nuclei are shown in blue (DAPI). Magnification of 40x, scale bar: 20 μm. *N* = 5.

We first isolated and cultured olfactory ensheathing glia (OEG) from the olfactory bulbs of adult rats following a protocol previously established by us and others (Nash et al., 2001; Carvalho et al., 2014, 2018). We also investigated if OEG in culture expresses components of the ECS, i.e., cannabinoid receptor type 1 (CB1), and the main endocannabinoid degradation enzymes, fatty acid amide hydrolase (FAAH) and monoacylglycerol lipase (MAGL), which degrade anandamide (AEA) and 2-arachidonoyl glycerol (2-AG), respectively, by qRT-PCR (Figure 1A). In addition, we showed that OEG primary cell cultures were positive for CB1, a membrane marker (Figures 1B, C), and MAGL, a cytoplasmic enzyme (Figure 1D).

To confirm OEG identity, an antibody against p75(NTR) was used as an OEG marker (Ramón-Cueto and Nieto-Sampedro, 1992). We also co-stained OEG cultures with antibodies against 2’,3’ cyclic nucleotide 3’-phosphodiesterase (CNPase) and smooth muscle α-actin (SMA). Our cultures were double positive for all used markers and displayed a total of 95-99% p75(NTR)-positive cells, confirming OEG identity and purity (Jahed et al., 2007; Higginson and Barnett, 2011; Figures 1B-D).

### 3.2. OEGs cultures produce endocannabinoids and related mediators

**FIGURE 2:**
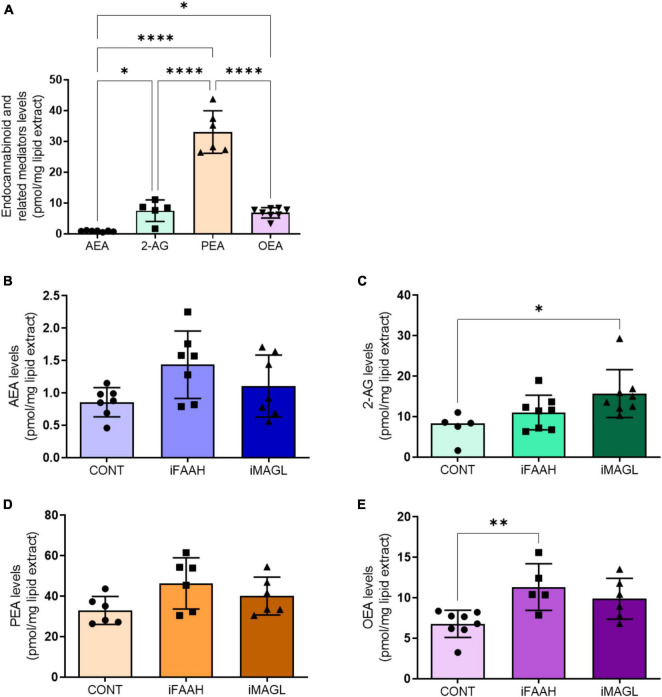
OEG cell cultures release endocannabinoids to OEGCM. In panel **(A)**, anandamide (AEA, *N* = 7) and 2-arachidonoylglycerol (2-AG, *N* = 5), and related mediators palmitoylethanolamide (PEA, *N* = 6) and oleoylethanolamide (OEA, *N* = 8) levels in the olfactory ensheathing glia conditioned medium (OEGCM) (*p* = 0.0368 for AEA vs. 2-AG; *p* < 0.0001 for AEA vs. PEA; *p* = 0.0334 for AEA vs. OEA; *p* < 0.0001 for 2-AG vs. PEA; *p* = 0.9998 for 2-AG vs. OEA; *p* < 0.0001 for PEA vs. OEA). In panels **(B–E)**, levels of endocannabinoids and related mediators after treatment with URB597 10^– 9^ M (iFAAH) or JZL184 10^– 9^ M (iMAGL) for 3 days before lipid analysis. Data is presented as mean ± SD, statistical analysis was performed using One-way ANOVA and Tukey’s as *post hoc* test. **(B)** *N* = 7 for all experimental groups, *p* = 0.0527 for CONT vs. iFAAH, *p* = 0.5351 for CONT vs. iMAGL; **(C)** Control: *N* = 5, iFAAH: *N* = 8, iMAGL: *N* = 8, *p* = 0.4139 for CONT vs. iFAAH, *p* = 0.0202 for CONT vs. iMAGL; **(D)** *N* = 6 for all experimental groups, *p* = 0.0817 for CONT vs. iFAAH, *p* = 0.4486 for CONT vs. iMAGL; **(E)** Control: *N* = 8, iFAAH: *N* = 5, iMAGL: *N* = 6, *p* = 0.0084 for CONT vs. iFAAH, *p* = 0,0587 for CONT vs. iMAGL. *p < 0.05, **p < 0.01, and ****p < 0.0001.

To determine whether OEGs can produce endocannabinoids, we quantified the levels of AEA and 2-AG, and the AEA related mediators, palmitoylethanolamide (PEA) and oleoylethanolamide (OEA), in the OEGCM. As shown, OEGCM presents higher PEA concentrations than AEA, 2-AG or OEA (Figure 2A and Table 2). To investigate what enzymes regulate endocannabinoid or related mediator levels in OEGCM, OEGs were cultured with URB597 10^–9^ M (iFAAH), a selective inhibitor of FAAH, or with JZL184 10^–9^ M (iMAGL), a selective inhibitor of MAGL, for 72h before lipid analysis. As shown in Figure 2, in the presence of iFAAH, the levels of AEA (control: 0.8571 ± 0.2254, iFAAH: 1.436 ± 0.5208, iMAGL: 1.106 ± 0.4785, Figure 2B) only tended to increase, while in the presence of iMAGL, the levels of 2-AG were significantly increased compared to control cells (control: 7.478 ± 3.497, iFAAH: 11.05 ± 4.256, iMAGL: 15.72 ± 5.898, Figure 2C). Regarding the AEA related mediators, the levels of PEA did not show any difference from the control when treated with iFAAH or iMAGL (control: 33.00 ± 6.910, iFAAH: 46.3 ± 12.68, iMAGL: 40.12 ± 9.349, Figure 2D), while those of OEA were significantly increased (control: 6.793 ± 1.674, iFAAH: 11.34 ± 2.882, iMAGL: 9.893 ± 2.522, Figure 2E).

**TABLE 2:** Endocannabinoids and related mediator levels in olfactory ensheathing glia conditioned medium (OEGCM).

**Endocannabinoids and related mediators levels in pmol per mg of lipid extract (mean ± SD)**	
AEA	0.8571 @ 0.2254
2-AG	7.478 @ 3.497
PEA	33.00 @ 6.910
OEA	6.793 @ 1.674

### 3.3. Oligodendrocyte maturation is modulated by the presence of AM251 in hippocampal mixed cell cultures

**FIGURE 3:**
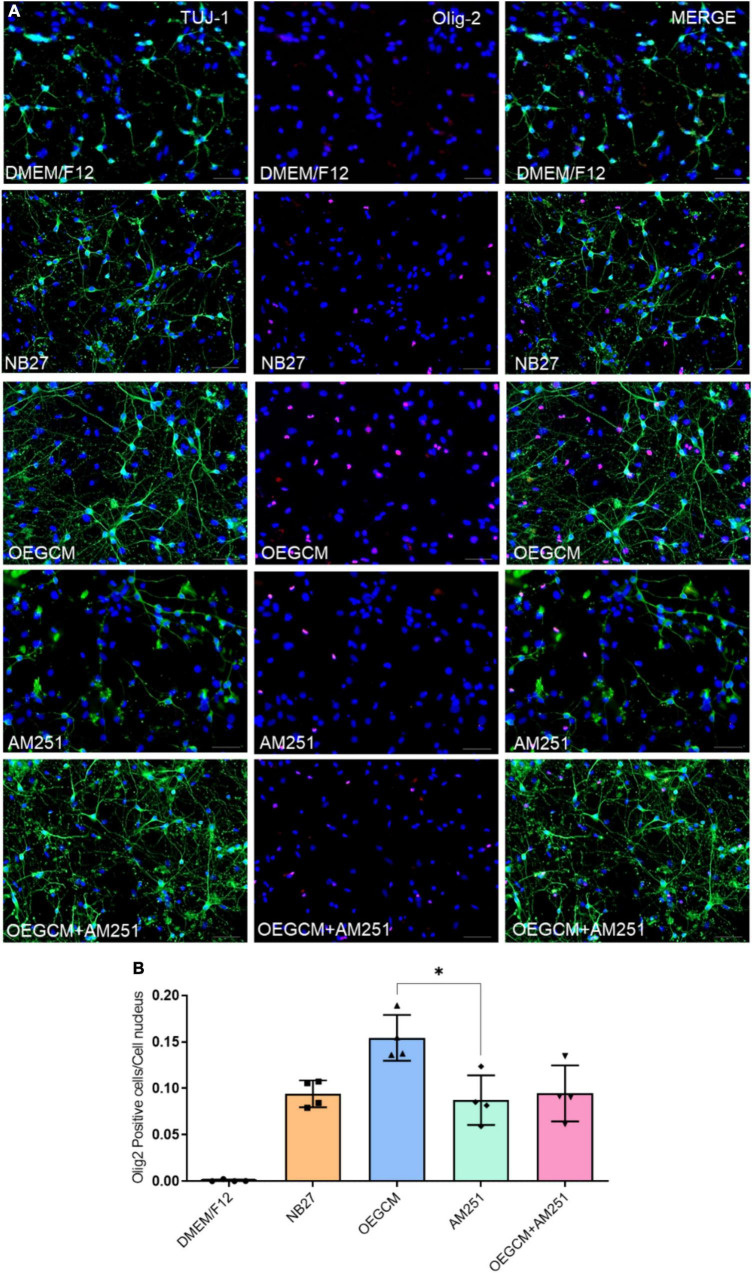
Addition of AM251 did not reduce the number of Olig2 positive cells increased by OEGCM in hippocampal cells in culture. In panel **(A)**, neurons labeled in green (TUJ-1), OLs in red (Olig2) and cell nuclei are shown in blue (DAPI); magnification of 20x, scale bar: 50 μm. In panel **(B)**, the number of OLs was evaluated counting the number of Olig2 positive cells in each condition (for each experiment, 5 images from different areas for each condition were used for the quantification). Data is presented as mean ± SD, statistical analysis was performed using Kruskal-Wallis and Dunn’s as *post hoc* test. *N* = 4 for all experimental conditions, *p* = 0.1066 for NB27 vs. OEGCM, *p* > 0.9999 for NB27 vs. AM251, *p* = 0.1564 for OEGCM vs. OEGCM + AM251, *p* > 0.9999 for AM251 vs. OEGCM + AM251, *p* = 0,0375 for OEGCM vs. AM251. *p < 0.05.

OL lineage differentiation is regulated by several transcription factors, including Olig1 and Olig2, known as major players in both embryonic and adult oligodendrogenesis and CNS myelination (Jakovcevski et al., 2009; Meijer et al., 2012). Olig2 is present at each oligodendroglial stage, from progenitor to mature cells, and is widely used as an OL marker (Marinelli et al., 2016). Our group previously showed that OEGCM increases the number of oligodendrocytes in hippocampal mixed-cell cultures (Carvalho et al., 2014). Here, we evaluated the potential of CB1 to mediate the OEGCM proliferative effect in Olig2-positive cells (Figure 3A). The statistical analysis however, did not show neither a significant increase in the number of Olig2-positive cells when treated with OEGCM, nor a significant decrease in the number of cells when treated with OEGCM + AM251 (DMEM/F12: 0.0008 ± 0.001217, NB27: 0.09407 ± 0.01446, OEGCM: 0.1544 ± 0.02473, AM251: 0.0874 ± 0.02673, OEGCM + AM251: 0.0946 ± 0.03013, Figure 3B).

**FIGURE 4:**
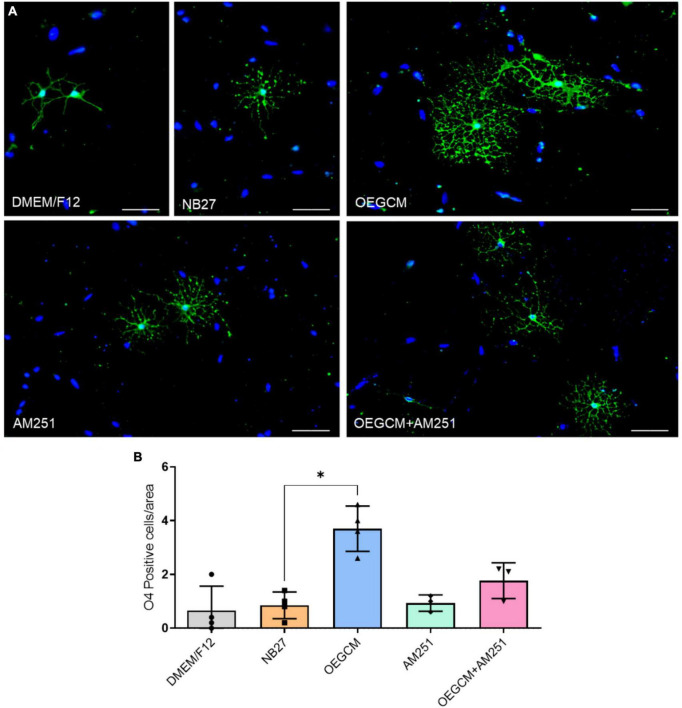
CB1 receptor blockade tends to decrease the O4-positive cells in hippocampal cells in culture. In panel **(A)**, representative immunocytochemistry of O4 oligodendrocytes in green and cell nuclei in blue (DAPI). Magnification of 20x, scale bar: 50 μm. In panel **(B)**, the number of oligodendrocytes in hippocampal cell culture was evaluated counting the number of O4-positive cells in each condition (for each experiment, 5 images from different areas for each condition was used for the quantification). Data is presented as mean ± SD, statistical analysis was performed using Kruskal-Wallis and Dunn’s as *post hoc* test. DMEM/F12: *N* = 4; NB27: *N* = 4; OEGCM: *N* = 4; AM251: *N* = 3; OEGCM + AM251: *N* = 3; *p* = 0.0254 for NB27 vs. OEGCM, *p* > 0.9999 for NB27 vs. AM251, *p* = 0.7858 for OEGCM vs. OEGCM + AM251, *p* > 0.9999 for AM251 vs. OEGCM + AM251, *p* = 0.0690 for OEGCM vs. AM251. *p < 0.05.

**FIGURE 5:**
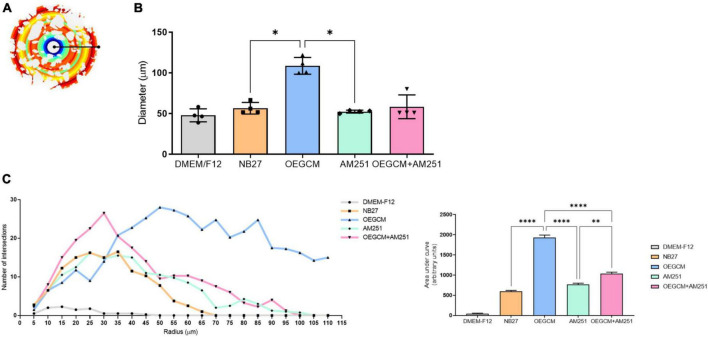
Sholl analysis from O4 data imply a role for CB1 on OEGCM ability to increase the complexity of oligodendrocytes process branching. In panel **(A)**, the Sholl circles from one O4-positive oligodendrocyte from OEGCM condition. In panel **(B)**, the diameter from O4-positive oligodendrocytes in the different conditions. Data is presented as mean ± SD, statistical analysis was performed using One-way ANOVA and Holm-Sídák’s as *post hoc* test. *N* = 4 for all experimental conditions, *p* = 0.0165 for NB27 vs. OEGCM, *p* = 0.8079 for NB27 vs. AM251, *p* = 0.0951 for OEGCM vs. OEGCM + AM251, *p* = 0.9585 for AM251 vs. OEGCM + AM251, *p* = 0.0121 for OEGCM vs. AM251. In panel **(C)**, the area under the curve calculates the number of intersections in every 0.5 μm radium in each Sholl circle. Data is presented as mean ± SD, statistical analysis of AUC was performed using One-way ANOVA and Sídák’s as *post hoc* test. *N* = 4 for all experimental conditions, *p* < 0.0001 for NB27 vs. OEGCM, *p* = 0.0586 for NB27 vs. AM251, *p* < 0.0001 for OEGCM vs. OEGCM + AM251, *p* = 0.0021 for AM251 vs. OEGCM + AM251, *p* < 0.0001 for OEGCM vs. AM251. *p < 0.05, **p < 0.01, and ****p < 0.0001.

OPCs and mature OLs express the surface antigen O4 as shown at hippocampal culture day 3 (Figure 4A). O4-positive cells have been commonly used as the earliest specific marker recognized for oligodendroglia lineage, enabling the recognition of the pre-myelination stage, or pre-oligodendrocytes (Gomez et al., 2010; Goldman and Kuypers, 2015). In a pioneer study, Gomez and coworkers showed that CB1 and CB2 agonists can enhance the number of O4-positive cells in purified oligodendrocyte progenitor cell culture. Therefore, we went on to evaluate the effect of the OEGCM at promoting the proliferation of O4-positive cells. As shown in Figure 4B, we found that OEGCM significantly increases the number of O4-positive cells, but the addition of AM251 was not capable to show a significant decrease in the effect of OEGCM (DMEM/F12: 0.65 ± 0.9147, NB27: 0.85 ± 0.5, OEGCM: 3.7 ± 0.8406, AM251: 0.9333 ± 0.3055, OEGCM + AM251: 1.767 ± 0.6658). We then decided to evaluate the oligodendrocyte morphology in O4-positive cells in the presence of OEGCM (Figures 5A–C). We showed there was an increase in O4-positive cells process branching complexity, while treatment with AM251 reversed this effect, as shown with the assessment of the number of intersections in every 0.5 μm in each concentric circle going from the soma to the periphery, demonstrated through the area under the curve, where the complexity of the oligodendrocyte branching is directly proportional to the number of intersections (DMEM/F12: 47.5 ± 23.16, NB27: 598.1 ± 46.29, OEGCM: 1934.0 ± 117.5, AM251: 765.6 ± 71.76, OEGCM + AM251: 1036.0 ± 67.8, Figure 5C).

**FIGURE 6:**
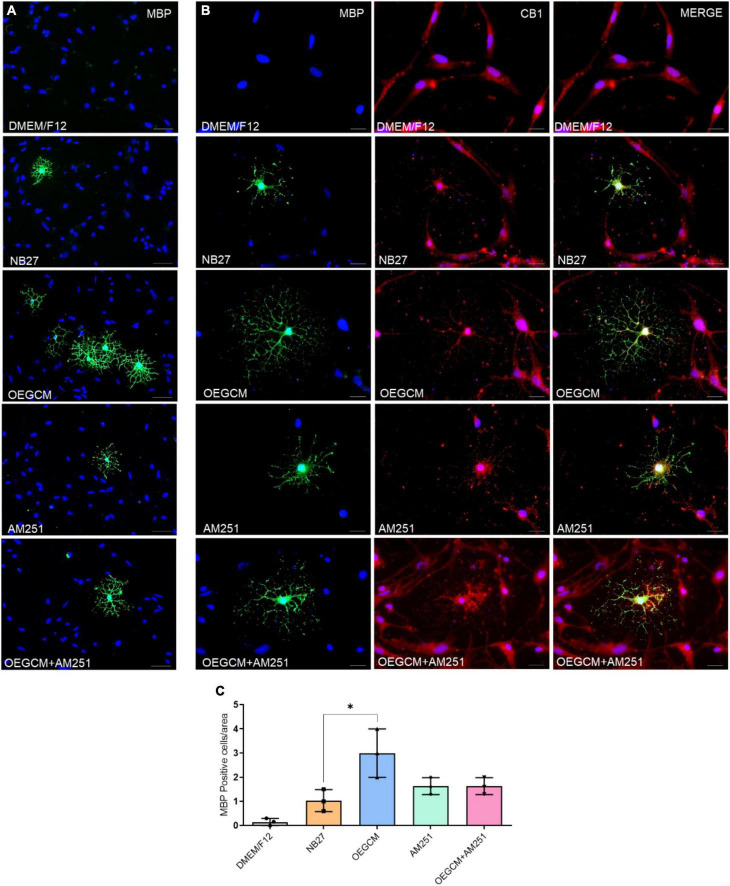
The blockade of CB1 receptor tends to drive a decrease in MBP expression in oligodendrocytes in hippocampal cells in culture. In panel **(A)**, MBP positive cells are shown in green and in blue, the cell nuclei (DAPI). Magnification of 20x, scale bar: 50 μm. N = 3. In panel **(B)**, immunocytochemistry for MBP in green, CB1 in red and cell nuclei in blue (DAPI). Magnification of 20x, scale bar: 50 μm. *N* = 3. In panel **(C)**, the number of oligodendrocytes in hippocampal cell culture was evaluated counting the number of MBP-positive cells in each condition (for each experiment, 5 images from different areas for each condition was used for the quantification). Data is presented as mean ± SD, statistical analysis was performed using Kruskal-Wallis and Dunn’s as *post hoc* test. *N* = 3 for all experimental conditions, *p* = 0.0301 for NB27 vs. OEGCM, *p* > 0.9999 for NB27 vs. AM251, *p* = 0.6842 for OEGCM vs. OEGCM + AM251, *p* > 0.9999 for AM251 vs. OEGCM + AM251, *p* = 0.6842 for OEGCM vs. AM251. *p < 0.05.

**FIGURE 7:**
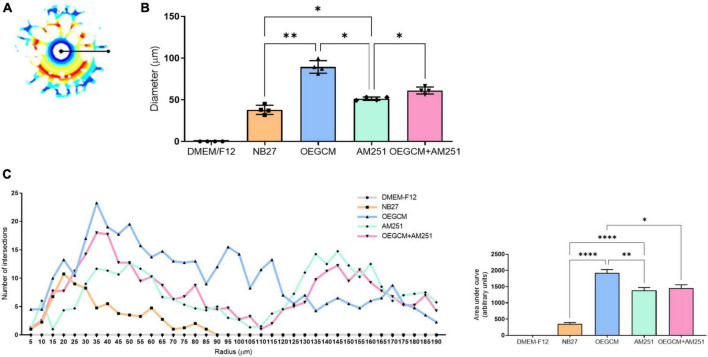
Sholl analysis from MBP immunocytochemistry shows that CB1 blockade decreases process branching complexity of oligodendrocytes stimulated by OEGCM. In panel **(A)**, the Sholl circles from one MBP positive oligodendrocyte from OEGCM condition. In panel **(B)**, the diameter from MBP positive oligodendrocytes in the different conditions. Data is presented as mean ± SD, statistical analysis of AUC was performed using One-way ANOVA and Sídák’s as *post hoc* test. *N* = 4 for all experimental conditions, *p* = 0.0024 for NB27 vs. OEGCM, *p* = 0.0427 for NB27 vs. AM251, *p* = 0.0568 for OEGCM vs. OEGCM + AM251, *p* = 0.0472 for AM251 vs. OEGCM + AM251, *p* = 0.0101 for OEGCM vs. AM251. In panel **(C)**, the area under the curve calculates the number of intersections in every 0.5 μm radium in each Sholl circle. Data is presented as mean ± SD, statistical analysis of AUC was performed using One-way ANOVA and Sídák’s as *post hoc* test. *N* = 4 for all experimental conditions, *p* < 0.0001 for NB27 vs. OEGCM, *p* < 0.0001 for NB27 vs. AM251, *p* = 0.0117 for OEGCM vs. OEGCM + AM251, *p* = 0.9831 for AM251 vs. OEGCM + AM251, *p* = 0.0040 for OEGCM vs. AM251. *p < 0.05, **p < 0.01, and ****p < 0.0001.

We also decided to evaluate the MBP-positive cells in hippocampal mixed cultures cultured (or not) with the OEGCM, in the presence of the CB1 antagonist. MBP is one of the main myelin components, composed of several plasmatic membrane layers from OLs, and appears during myelination or remyelination steps, and later stages of OL differentiation (Meffre et al., 2015). We performed a CB1 co-staining in the MBP-positive cells to confirm the expression of this receptor in OLs (Figures 6A, B). We found that OEGCM increased MBP-positive cells (DMEM/F12: 0.1533 ± 0.1501, NB27: 1.033 ± 0.4509, OEGCM: 3.000 ± 1.000, AM251: 1.633 ± 0.3512, OEGCM ± AM251: 1.633 ± 0.3512, Figure 6C) and OL size and arborization, assessed by the diameter of the OL (DMEM/F12: 0.0000 ± 0.0000, NB27: 37.94 ± 5.491, OEGCM: 89.38 ± 7.592, AM251: 51.31 ± 1.951, OEGCM ± AM251: 61.13 ± 4.194, Figures 7A, B) and number of intersections in each 0.5 mm from soma to periphery (Figure 7C). We also observed that the addition of AM251 was able to decrease the process branching complexity of the OL promoted by the OEGCM when evaluating the number of intersections at each radium evaluated (Figure 7C). These results indicate that the additional effects of OEGCM in O4 and MBP-positive cells could be mediated by CB1 activation.

### 3.4. Endocannabinoids modulate the branching complexity of premyelinating and myelinating oligodendrocytes in hippocampal cells in cultures

**FIGURE 8:**
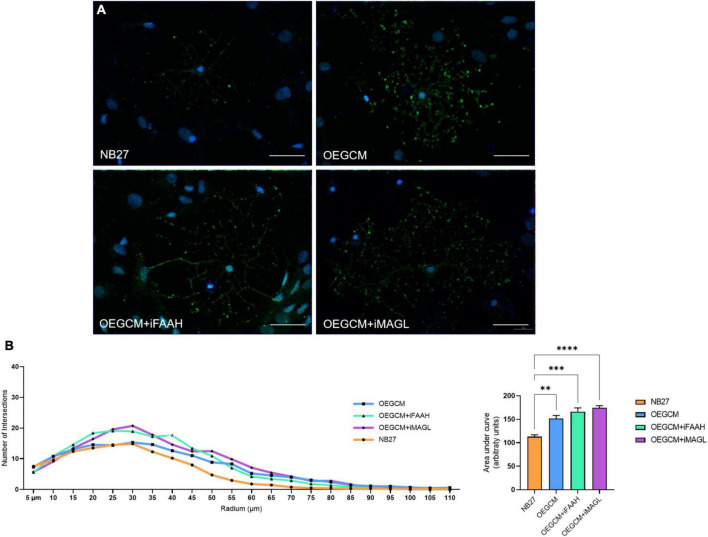
OEGCM from cultures treated with FAAH or MAGL inhibitors do not exert effects over the complexity of oligodendrocytes process branching assessed by O4 marker. In panel **(A)**, representative immunocytochemistry of the O4-positive cells in green and cell nuclei in blue (DAPI). Magnification of 40x, scale bar: 20 μm. In panel **(B)**, the area under the curve calculates the number of intersections in every 0.5 μm radium in each Sholl circle. Data is presented as mean ± SD, statistical analysis of AUC was performed using One-way ANOVA and Tukey’s as *post hoc* test. *N* = 4 for all experimental conditions, *p* = 0.0030 for NB27 vs. OEGCM, *p* = 0.0002 for NB27 vs. OEGCM + iFAAH, *p* < 0.0001 for NB27 vs. OEGCM + iMAGL, *p* = 0.3240 for OEGCM vs. OEGCM vs. OEGCM + iFAAH, *p* = 0.0733 for OEGCM vs. OEGCM + iMAGL. **p < 0.01, ***p < 0.001, and ****p < 0.0001.

**FIGURE 9:**
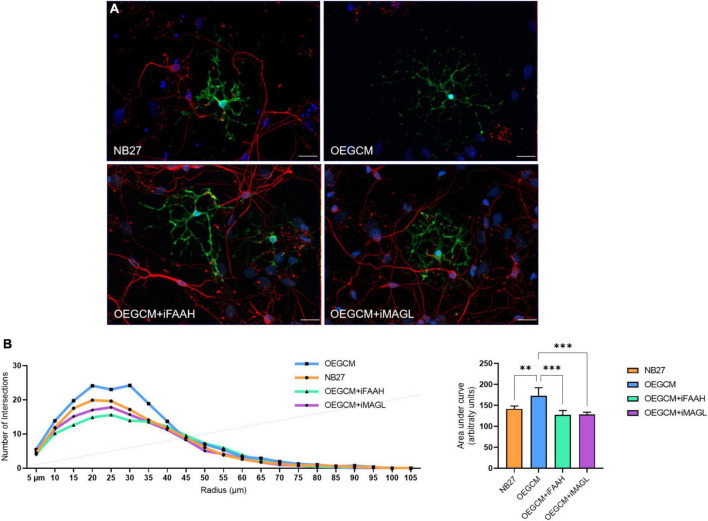
OEGCM from cultures treated with FAAH or MAGL inhibitors decreases the complexity of mature oligodendrocytes process branching. In panel **(A)**, representative immunocytochemistry of MBP-positive cells showed in green, neurons stained with TUJ-1 in red and cell nuclei shown in blue (DAPI). Magnification of 40x, scale bar: 20 μm. In panel **(B)**, the area under the curve calculates the number of intersections in every 0.5 μm radium in each Sholl circle. Data is presented as mean ± SD, statistical analysis of AUC was performed using One-way ANOVA and Tukey’s as *post hoc* test. NB27: *N* = 5, OEGCM: *N* = 3, OEGCM + iFAAH: *N* = 5, OEGCM + iMAGL: *N* = 4; *p* = 0.0061 for NB27 vs. OEGCM, *p* = 0.2070 for NB27 vs. OEGCM + iFAAH, *p* = 0.2750 for NB27 vs. OEGCM + iMAGL, *p* = 0.0003 for OEGCM vs. OEGCM + iFAAH, *p* = 0.0005 for OEGCM vs. OEGCM + iMAGL. **p < 0.01 and ***p < 0.001.

As shown, the selective inhibition of the degradation enzymes increases the concentration of CB1 ligand 2-AG and AEA related compounds (OEA) in the OEGCM. Therefore, we evaluated whether hippocampal mixed cell cultures incubated in the presence (or not) of OEGCM thus enriched with endocannabinoids could promote changes in the process branching complexity of the OL. We observed, through the analysis of the O4 surface antigen, that the OEGCM, in the presence of FAAH or MAGL inhibitors, did not promote an increase in the complexity of OL process branching when compared to the untreated conditioned medium (NB27: 113.0 ± 7.7, OEGCM: 151.4 ± 13.3, OEGCM + iFAAH: 166.4 ± 15.6, OEGCM + iMAGL: 174.4 ± 9.0, Figure 8). In contrast, the analysis of MBP showed that the treatment with the OEGCM from cells treated with either FAAH or MAGL inhibitors showed a decrease in the branching when compared to the cells treated with the OEGCM (NB27: 141.7 ± 7.0, OEGCM: 173.1 ± 19.2, OEGCM + iFAAH: 127.8 ± 10.1, OEGCM + iMAGL: 128.3 ± 5.6, Figure 9). These results suggest that the endocannabinoids may not add the same effect in the OEGCM trophic potential when comparing cells with different profiles of maturity, as the enhancement in the endocannabinoid content was not able to have a positive effect upon fully mature OLs.

### 3.5. The inhibition of MAGL and FAAH in OEG cell cultures and OEGCM does not affect the activation of the PI3K/Akt and ERK/MAPK signaling pathway in hippocampal mixed cells cultures

**FIGURE 10:**
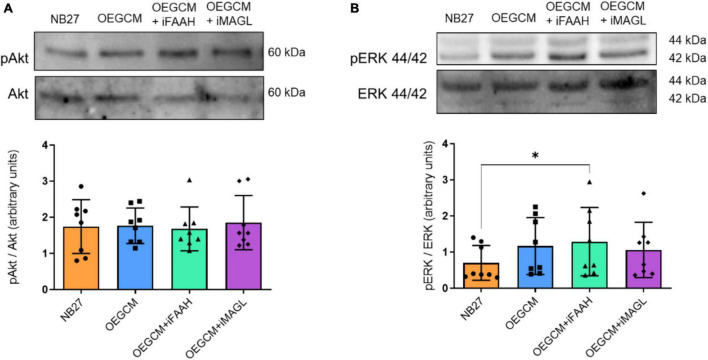
The phosphorylation of proteins associated with cellular signaling pathways in hippocampal cell cultures is not affected by the OEGCM from cells treated with FAAH or MAGL inhibitors. In panel **(A)**, representative western blotting of pAkt and Akt (60 kDa) and quantification of pAkt in relation to total Akt. Data is presented as mean ± SD, statistical analysis was performed using Friedman and Dunn’s as *post hoc* test. *N* = 8 for all experimental conditions, *p* > 0.9999 for all comparisons. In panel **(B)**, representative western blotting of pERK 44/42 and ERK 44/42 (44 kDa/42 kDa) and quantification of pERK 44/42 in relation with ERK 44/42. Data is presented as mean ± SD, statistical analysis was performed using Friedman and Dunn’s as *post hoc* test. *N* = 8 for all experimental conditions, *p* = 0.4882 for NB27 vs. OEGCM, *p* = 0.0402 for NB27 vs. OEGCM + iFAAH, *p* = 0.4882 for NB27 vs. OEGCM + iMAGL, *p* > 0.9999 for OEGCM vs. OEGCM + iFAAH, *p* > 0.9999 for OEGCM vs. OEGCM + iMAGL. *p < 0.05.

To complete our hypothesis that ECS could interfere in OEGCM trophic action on oligodendrocytes, we decided to investigate the phosphorylation of proteins responsible for the triggering of intracellular signaling pathways in hippocampal mixed cells cultured with the OEGCM, treated (or not) with inhibitors of FAAH and MAGL. More specifically, the phosphorylation of Akt and ERK 44/42 is known to be associated with the activation and consequent triggering of the signaling cascades that are important to oligodendrocyte maturation. No alterations were observed in the phosphorylation of either Akt (NB27: 1.74 ± 0.7436, OEGCM: 1.765 ± 0.4898, OEGCM + iFAAH: 1.679 ± 0.6068, OEGCM + iMAGL: 1.851 ± 0.7497, Figure 10A) or ERK44/42 (NB27: 0.700 ± 0,4825, OEGCM: 1.167 ± 0.7886, OEGCM + iFAAH: 1.288 ± 0.9478, OEGCM + iMAGL: 1.057 ± 0.7666, Figure 10B) when comparing the cultures that received modulated OEGCM to the cultures that received non-modulated OEGCM. These results show that the indirect modulation of the endocannabinoid content in OEGCM was not able to modulate the main signaling pathways involved with CB1 activation or oligodendrocyte development.

## 4. Discussion

In the present work, we demonstrate that OEG, a type of glia known to promote repair in the mammalian CNS (Ramón-Cueto and Munoz-Quiles, 2011), express components of the ECS and produce the main endocannabinoids, AEA and 2-AG, and AEA related mediators, PEA and OEA. CB1 is known to be one of the most abundant receptors in the CNS, found at high levels in the neocortex, hippocampus, basal ganglia, cerebellum, and brainstem (Kendall and Yudowski, 2016). However, the expression of CB1 as well as the role of the ECS in the olfactory system (Herkenham et al., 1991; Hutch et al., 2015) has been investigated only in the context of the control of olfaction (Breunig et al., 2010; Wang et al., 2012; Soria-Gómez et al., 2014).

In addition to the existence of the ECS and endocannabinoids in OEGs, our data demonstrate the active participation of the two main enzymes involved in endocannabinoid degradation, FAAH and MAGL. We observed that these cells can produce basal levels of endocannabinoids, and treatment of OEGs with specific inhibitors of these two enzymes elevated the levels of 2-AG and OEA, respectively, in OEGCM. The endocannabinoid 2-AG is mainly degraded by the action of MAGL, a cytoplasmic serine hydrolase, which in the brain accounts for approximately 75% of the degradation of 2-AG to arachidonic acid (AA) and glycerol (Ueda et al., 2013). OEA, on the other hand, is a *N*-acylethanolamine (NAE), like AEA and PEA. The degradation of NAEs occurs mainly through the action of FAAH, a serine hydrolase associated with the inner membrane of the cells (Cravatt et al., 1996). In addition to FAAH, NAEs can be actively degraded by other routes, which include cyclooxygenase 2 (COX2) and lipoxygenases (LOX) (Rodriguez de Fonseca et al., 2005; Ueda et al., 2013), and by other hydrolases, such as *N*-acylethanolamine acid amidohydrolase (NAAA) (Ueda et al., 2010). Our data show an increase in the levels of the OEA, but not of AEA or PEA, following incubation with the FAAH inhibitor.

CB1 blockade through the addition of AM251 altered the ability of the olfactory ensheathing glia conditioned medium to increase the maturation of oligodendrocytes in rat hippocampal neuron mixed cell cultures, such trophic potential already shown by Carvalho and collaborators (Carvalho et al., 2014). Our data strengthen the idea that activation of CB1 receptors positively modulate OL differentiation (Solbrig et al., 2010; Gomez et al., 2011, 2015). Regarding OL proliferation, activation of CB1 provides a direct link to Olig2 expression in the rat SVZ, as shown in *in vivo* experiments (Arevalo-Martin et al., 2007), while CB2 activation increases polysialylated neural cell adhesion in the same area. During modulation of oligodendrogenesis, the expression of specific transcription factors is necessary, such as the basic-helix-loop-helix transcription factor Olig2, which is one of the key markers present in OPCs, located in the nucleus of mature OLs (Xapelli et al., 2014; Marinelli et al., 2016). Although we were not able to show a statistically significant decrease in the number of Olig2-positive cells in hippocampal cell cultures, we do see that there is a tendency in the AM251 in reversing this increase. We also show that the addition of AM251 is capable of impairing the complexity of the OL process branching promoted by OEGCM.

It has been proposed that cannabinoid receptors modulate OL maturation and alter their morphology, with CB1/CB2 activation being able to increase the process branching complexity in these cells (Gomez et al., 2010, 2011). Indeed, Win 55,212-2, a non-selective CB1/CB2 agonist, promoted an increase in O4-positive cells after a demyelinating condition in rats (Solbrig et al., 2010), an effect mediated by CB1/CB2 activation and depending on the activation of ERK/MAPK, PI3K/Akt and mTOR pathways (Gomez et al., 2010, 2011). Our results related to pre-OLs show a decrease in O4-positive cell process branching complexity in the presence of AM251, suggesting that the activation of CB1 in OLs is a necessary step to OEGCM to exert its trophic potential.

The final step for OL maturation is the myelination stage, when myelin starts to be produced, creating the concentric layers of modified cell membranes that sheath axons (van Tilborg et al., 2018). Myelin proteins include the myelin oligodendrocyte glycoprotein (MOG), the myelin-associated glycoprotein (MAG) and the myelin basic protein (MBP) (Marinelli et al., 2016). Both CB1 and CB2 activation stimulate MBP expression on OLs, as shown using *in vivo* and *in vitro* approaches (Arevalo-Martin et al., 2007; Gomez et al., 2010, 2011). In our work, we observed that AM251 reduces the process branching complexity promoted by OEGCM, suggesting the importance of CB1 for complete OL maturation and the enhancement in the myelination stage induced by OEGCM. Unexpectedly, we also observed an increase in cell diameter and branching complexity in MBP-positive OLs when cultures were treated with AM251 alone, when compared with control. This effect could be explained by the fact that AM251 can also act as inverse agonist and, by itself, promote positive outcomes regarding neuronal development (Jin et al., 2004; Hill et al., 2006; Parihar et al., 2021). These results suggest a role for CB1 in determining their final morphological characteristics.

Considering that the CB1 receptor blockade diminished the trophic potential of the OEGCM over OL in the hippocampal mixed cell culture, we investigated if this trophic potential would be intensified with the increase of the endocannabinoid content in the conditioned medium, by treating the OEG cell culture with synthetic inhibitors of the degradation enzymes FAAH and MAGL. Our hypothesis was that, since the activation of the CB1 receptor is able to modulate OL development, the pharmacological enhancement of the concentrations of endocannabinoids could potentiate the maturation of OLs. By assessing the process branching complexity of OL, however, we observed no effect of the inhibitors when compared with the control conditioned medium, when OLs were assessed using the O4 marker. In contrast, the evaluation of MBP, which characterizes a full mature OL, showed that enhancing the content of endocannabinoids and related mediators in OEGCM was able to diminish the branching complexity of the cells, when compared to OEGCM only. This modulation of the endocannabinoid system in a cell such as the OL is unprecedented in the literature. The use of an indirect way as the one described in this work, through the increase in the endocannabinoid content in a conditioned medium of a cell type that has the potential of modulating positively the morphology of OLs, however, depends on the concentration of the inhibitors used. It could be possible that the concentration of 10^–9^ M, although enough to increase the content of endocannabinoids and related molecules in the conditioned medium, was too low to allow a more efficient availability of endocannabinoids in the medium, and thus unable to activate as many receptors in an efficient way to induce a visible response. Regarding the effect over mature OLs, it is possible that such modulation could be able to activate other types of receptors, since FAAH and MAGL inhibitors increase the concentrations of AEA and 2-AG congeners acting at non-CB1 receptors. Experimental variables such as time of incubation could also impact this evaluation. Another hypothesis could be that the endocannabinoid system could be more involved in more immature stages of OL development than in more differentiated cells. It is interesting to highlight that this is the first study that evaluated the differentiation of OLs through indirect modulation of the endocannabinoid system in a mixed cell culture, in contrast with previous studies that used purified oligodendrocyte cultures (Gomez et al., 2011, 2015). In this way, we could assess the behavior of this type of glia in a microenvironment that has all types of interaction between all types of cells present in the CNS, mimicking in the most realistic manner possible the complexity of the system. However, we cannot rule out that other trophic factors, present in this conditioned medium, could exert their effects along with the endocannabinoids and endocannabinoid-like mediators up-regulated by FAAH and MAGL inhibitors, or counteract their action.

In a previous study, a positive effect of OEGCM in selectively generating OLs is dependent on the activation of selective intracellular pathways, such as PI3K/Akt, p38MAPK and ERK/MAPK, was shown (Carvalho et al., 2014). These intracellular pathways are downstream of the ECS in different cell tissues and modulated through distinct pharmacological tools (Sampaio et al., 2015; Iannotti et al., 2016). Other studies have shown the importance of the ECS in the modulation of OL proliferation and differentiation involving some of these pathways (Molina-Holgad et al., 2002; Huerga-Gómez et al., 2021), as seen, for example, for the ability of CB1 and CB2 agonists to promote OL differentiation through PI3K/Akt and the mammalian target of rapamycin (mTOR) activation (Gomez et al., 2011, 2015). Therefore, knowing that the activation of the cannabinoid receptors and the OL development share the same signaling pathways (Bouaboula et al., 1995; Galve-Roperh et al., 2002; Palazuelos et al., 2012; Gaesser and Fyffe-Maricich, 2016), we sought to evaluate if the treatment with different modulated conditioned mediums would be able to affect the phosphorylation of Akt and ERK 44/42. However, the change in the endocannabinoid content in these mediums did not affect the phosphorylation of Akt or ERK 44/42 when comparing the treatment groups with non-modulated OEGCM. This apparent non-modulation might be due to the fact that we are assessing the population of the OL in this culture as a whole, not by every stage of maturation, and also as discussed before, a possible low concentration of endocannabinoids able to exert cascading effects. We show in the experiments assessing the process branching complexity of the OLs that there are differences concerning the level of maturity of the cell. This, in addition to the presence of other cell types in the microenvironment, leads to an inability to see a significant effect in a single group of cells. Besides, we cannot rule out other signaling pathways involved in the oligodendrogenesis that were not investigated in this study, such as the p38MAPK pathway (Bhat et al., 2007; Chew et al., 2010), and which could be modulated instead of the pathways evaluated here.

Demyelinating injuries, such as multiple sclerosis, acute disseminated encephalomyelitis, leukodystrophy and Guillain-Barré’s syndrome, are some of the best-characterized diseases that occur due to loss or dysfunction of oligodendrocytes and myelin. Impairment in one or more steps of OL migration, proliferation, differentiation or maturation can stimulate the progress of these conditions (Marinelli et al., 2016; Rodrigues et al., 2017). There is no cure for demyelinating diseases to date, and the best treatment can only slow down the symptoms and/or the rate of demyelination. Due to the continued generation of oligodendrocytes from OPCs in some key areas of the CNS, the search for approaches that promote OL proliferation and differentiation into new and mature cells arouses interest in the scientific and medical fields (Rodrigues et al., 2017). Indeed, the application of both OEG and OEGCM is promising in preclinical studies not only in regeneration (Shyu et al., 2008; Myers et al., 2016) but also in cancer therapy (Carvalho et al., 2018). In addition to the unprecedented finding of CB1 involvement in OEGCM effects, our study suggests that OEGs could be a therapeutic target for endo- and phytocannabinoids (Russo and Marcu, 2017), with a focus on CB1 and MAGL. In addition, the ECS might regulate the composition of trophic activity present in OEGCM, as well as its ability to increase the branching complexity of OLs in hippocampal cell culture.

Our findings suggest that the ECS may be involved in the OEGCM composition and its influence on OL morphologic differentiation. Further studies should be carried out to unravel the connection between the trophic effects of OEG, the ECS and oligodendrogenesis, and maybe even using demyelinating pathology models *in vivo*.

## Data availability statement

The original contributions presented in this study are included in the article/supplementary material, further inquiries can be directed to the corresponding author.

## Ethics statement

This animal study was reviewed and approved by Ethics Committee for the Use of Animals in Research of the Federal University of Rio de Janeiro and in accordance with the guidelines of the Brazilian Society of Neuroscience and Behavior.

## Author contributions

ME-L, SA, FI, VM, and BT: conception and design, provision of study material, collection and assembly of data, data analysis and interpretation, manuscript writing, and final approval of the manuscript. YP-C, PT, LV, FP, AI, RS-S, AA, and RC: collection and assembly of data, and data analysis and interpretation. FM, LS, and RD: conception and design, provision of study material, assembly of data, data analysis and interpretation, manuscript writing, final approval of manuscript, and financial support and administrative support. All authors contributed to the article and approved the submitted version.
